# Virus-Based Nanomaterials and Nanostructures

**DOI:** 10.3390/nano10030567

**Published:** 2020-03-20

**Authors:** Jin-Woo Oh, Dong-Wook Han

**Affiliations:** 1 Department of Nanofusion Technology, College of Nanoscience & Nanotechnology, Pusan National University, Busan 46241, Korea; 2 Department of Cogno-Mechatronics Engineering, College of Nanoscience & Nanotechnology, Pusan National University, Busan 46241, Korea

**Keywords:** virus-based nanomaterials, energy devices, biomedical applications, self-assembly, piezoelectric biomaterials

## Abstract

This Special Issue highlights the recent developments and future directions of virus-based nanomaterials and nanostructures in energy and biomedical applications. The virus-based biomimetic materials formulated using innovative ideas presented herein are characterized for the applications of biosensors and nanocarriers. The research contributions and trends based on virus-based materials, covering energy-harvesting devices to tissue regeneration over the last two decades, are described and discussed.

Virus-based biomimetic materials derived from plant viruses and bacteriophages rarely generate harmful side effects in human beings since phages do not consist of mammalian promoter sequences in their genomes [1]. The structures of viruses consist of two or three parts: (1) the genetic material composed from either DNA or RNA, which carries genetic information; (2) a protein coat, called the capsid, which surrounds and protects the genetic material; and in some cases (3) an envelope of lipids that surrounds the protein coat [2]. Advantageously, the monodispersed phages can self-assemble to develop rope-like bundles or liquid crystals and their surface can be modified either by chemical or genetic modifications [1,3]. Due to these unique properties, many research findings based on phage-based biomaterials have been developed for applications in drug delivery, biosensors, biomedical imaging, tissue regeneration, energy, and catalysis [4,5].

The current Special Issue, including 11 original research works, focuses on highlighting the progress, challenges, and future directions in the area of virus-based nanomaterials and nanostructures with multiple applications in biomedicine and energy. Researchers have studied mineralization, magnetization, bioconjugation, and drug delivery for the development of biosensors and vaccines [6,7,8,9,10,11,12]. Research findings of virus-based biomimetic materials in energy, biosensors, and tissue regeneration over the last two decades are comprehensively discussed in reviews [13,14,15,16].

Kim et al. propose an M13-bacteriophage-based colorimetric multi-array biosensor that can classify four different antibiotics (duricef, citopcin, amoxicillin, and rifampin) and hormone (estrogen) drugs including mercilon, gestodene, estrone, and estradiol by analyzing the color change [6]. The sensor can be fabricated using self-assembly of genetically engineered M13 bacteriophages, which incorporates peptide libraries on its surface. The fabricated sensor platform consists of a sensor chip that is 1 cm^2^ wide, a chamber of about 30 cc capacity, and a small webcam. This biosensor system is inexpensive and easy to apply in monitoring the environment and health care. The color change in the biosensor is caused by a reaction between the sensor array and external substances, detected by a complementary metal–oxide–semiconductor detector, and followed by employing hierarchical cluster analysis.

Yuste-Calvo et al. developed turnip mosaic virus (TuMV)-based nanoparticles to detect antibodies with high sensitivity [7]. TuMV is a virion with an elongated and flexuous structure. It is 700 nm long and 12 nm wide. Nearly 2000 copies of coat protein are present in each particle. The research group modified TuMV virus-like particles (VLPs) with a peptide from the chaperonin Hsp60, which is known to be involved in inflammation processes and autoimmune diseases. The quantitative detection of anti-Hsp60 autoantibodies is demonstrated through the multimeric presentation of the epitopes on TuMV VNPs through an in vivo murine (adult C57BL/6J) model. In particular, the high sensitivity of the developed Hsp60-VLPs provides a novel effective tool for diagnosis, progression, and prognosis in inflammation-mediated disorders. The model is suggested to reproduce the clinical, histopathological, and immune characteristics observed in humans by the induction of chronic colitis associated with diarrhea and weight loss.

Damm et al. functionalized the surface of calcium phosphate (CaP) nanoparticles with stabilized trimers of the HIV-1 envelope (Env), resulting in Env-CaP-p30 nanoparticles, to demonstrate improvement of Env antibody responses by intrastructural help (ISH) [8]. The Env trimers (MW = 140 kDa) are coupled to the nanoparticle surface using sulfosuccinimidyl-trans-4-(N-maleimidomethyl)cyclohexane-1-carboxylate (sulfo-SMCC) cross-linker, which reacts primary amines in Env with the thiol groups on the nanoparticle surface. The in vitro studies explored the Env-CaP-p30 nanoparticles’ induction of the activation of naive Env-specific B-cells in contrast to soluble Env trimers. The authors applied the nanoparticles to study the effects of ISH in mice immunized with a licensed vaccine against tetanus toxoid.

Atanasova et al. determined whether the surface hydrophilicity of tobacco mosaic virus (TMV) particles can be manipulated through covalent attachment of polymer molecules [9]. Three different polymers, namely perfluorinated poly(pentafluorostyrene) (PFS), the thermo-responsive poly(propylene glycol) acrylate (PPGA), and the block copolymer polyethylene-block-poly(ethylene glycol), were examined. In this study, wild-type tobacco mosaic virus (wt-TMV) and a TMV-cysteine (Cys) mutant presenting thiol groups help with the covalent binding of the organic molecules. The covalent attachment makes the virus surface hydrophobic without affecting the integrity of the capsid and suppresses the virus mineralization by inorganic deposits. The growth mechanism of the inorganic material on the virus surface was analyzed in terms of the optical properties, bandgap (Eg), and particle size of solution-grown zinc sulfide (ZnS) nanoparticles. The authors concluded from the ZnS mineralization test that the degree of the virus hydrophobicity can be tuned by the polymer properties.

Suhaimi et al. developed a vaccine by expressing *Neospora caninum* profilin (NcPROF) in silkworm larvae by recombinant *Bombyx mori* nucleopolyhedrovirus (BmNPV) bacmid [10]. They investigated three NcPROF-based constructs for the recombination viz. native NcPROF fused with an N-terminal protective antigen (PA)tag (PA-NcPROF), PA-NcPROF with the signal sequence of bombyxin from *B. mori* (bx-PA-NcPROF), and bx-PA-NcPROF with additional C-terminal transmembrane and cytoplasmic domains of GP64 from BmNPV (bx-PA-NcPROF-GP64TM). Only bx-PA-NcPROF-GP64TM was found to be secreted as nanoparticles with binding affinity to its receptors and pelleted, whereas the remaining two were not.

Rybka et al. characterized the assembly of hepatitis B virus capsid protein into virus-like particles (HBc VLPs) with the magnetic core of superparamagnetic iron oxide nanoparticles (SPIONs) [11]. The synthesized SPIONs were functionalized with two different ligands—1,2-,istearoyl-sn-glycero-3-phosphoethanolamine-N-[carboxy-(polyethyleneglycol)] (PL-PEG-COOH) or dihexadecyl phosphate (DHP)—to further assess crucial parameters guiding SPION-HBc VLP assembly. They evaluated the mechanism of self-assembly as well as the antigenicity of SPION-HBc VLPs.

Finbloom et al. investigated three morphologically distinct VLPs, namely a 27 nm MS2 sphere, an 18 nm tobacco mosaic virus (TMV) disk, and a 50 nm nanophage filamentous rod conjugated to doxorubicin (DOX), for their drug delivery potential to glioblastoma [12]. Although all VLPs exhibited adequate drug delivery and cell uptake in vitro, the authors found that the survival rates of glioma-bearing mice treated with TMV showed the best responses in vivo. The results of physicochemical and biological characterizations suggested that these VLPs can be promising nanocarriers for the convection-enhanced delivery (CED) of traditional chemotherapeutics such as DOX in glioblastoma treatment.

Shin et al. provide a comprehensive review of the principles, properties, and role of organic piezoelectric biomaterials in energy and biomedical applications [13]. The biomedical devices featuring the biocompatible piezoelectric materials are useful in energy-harvesting devices, sensors, and scaffolds for cell and tissue engineering. The review addresses how to tackle issues related to the better integration of organic piezoelectric biomaterials into biomedical devices. When mechanically agitated, piezoelectric materials accumulate an electric charge. Although organic piezoelectric biomaterials possess weak piezoelectricity compared with their inorganic counterparts, they can serve as the functional materials in the field of medically mountable and implantable applications when they are well processed. Piezoelectric mechanisms and the properties of the materials, including collagen, glycine, M13 bacteriophage, silk, cellulose, diphenylalanine, and poly(vinylidene fluoride), are discussed throughout the review.

Park et al. describe methods to fabricate M13-bacteriophage-based piezoelectric energy-harvesting devices to develop high-performance and biocompatible energy devices for a wide range of practical applications [14]. Due to surface modification, M13 bacteriophages overcome other natural biomaterials with limitations in mass production and low piezoelectric properties. M13 bacteriophages exhibit unique features such as similar structures with collagens, mass amplification, genetic modification, liquid–crystalline phase transition, and excellent piezoelectric properties, which distinguish them from other materials. Among the M13-phage-based piezoelectric energy-harvesting devices, vertically aligned phage films exhibited the highest performance with a peak voltage of 2.8 V and a peak current of 120 nA. The review suggests some strategies, such as fabricating triboelectric devices based on M13 phages and developing composite structures composed of organic and inorganic biomaterials, to enhance the power of devices.

In another review, Moon et al. highlight the recent progress made in the application of M13-bacteriophage-based sensor systems and discuss future M13 bacteriophage technology [15]. Genetic engineering provides many possibilities to use M13 bacteriophages as the core material of sensors. Due to this engineering, these bacteriophages exhibit specific binding affinity to target materials, including chemicals and biological materials. The modification mainly occurs on the pVIII protein of the M13 bacteriophage. M13 bacteriophages display similar structure and behavior to liquid crystals during fabrication. The crystal structure of self-assembled M13 bacteriophages exhibits different phases, i.e., nematic, cholesteric, and smectic at low, medium, and high concentrations, respectively. Using a natural M13 bacteriophage without any genetic engineering also provides the opportunity to interact with ligands due to an abundant negative charge on the surface protein. The charge distribution of the C-terminus (positive) and N-terminus (negative) induces a strong dipole to M13 bacteriophages providing a natural negative charge, which enables them to interact with positively charged materials including carbon nanofibers. The sensing potential of M13 bacteriophages toward proteins, microorganisms, chemicals, and color is discussed throughout this review.

Raja et al. describe the emerging trend of virus-based biomimetic materials in tissue regeneration [16]. The review outlines the integration of the virus into biomaterials with different morphologies such as hydrogels, two-dimensional substrates, and nanofibers in the field of tissue regeneration. It discusses remarkable properties of medicinally valued viruses including fd, M13, TMV, T7, and Potato virus X (PVX). Apart from tissue regeneration, the review covers other biomedical applications such as drug delivery, bioimaging, and biosensing. In conclusion, the authors recommend a systematic study using virus-based biomimetic materials to explain the different phases of tissue regeneration. Sophisticated techniques and methodologies are required to estimate the number of peptides expressed on each phage particle. Nonetheless, many viral nanocomposites in the form of polymeric micelles, vesicles, and dendrimers remain to be developed.

In conclusion, as the Editors of this Special Issue, we would like to thank all the authors and reviewers, who contributed to this Special Issue with innovative ideas and constructive reviewers’ comments. We would like to express appreciation for the consistent support from the Editorial Office. We are sure that this Special Issue will provide our readers with a platform to understand the advantageous properties of benevolent virus-based nanomaterials and nanostructures with their pivotal roles in energy and biomedical applications.
